# Type D Personality Is an Independent Predictor of Lower Urinary Tract Symptoms in Young Men

**DOI:** 10.3389/fpsyg.2022.822490

**Published:** 2022-02-22

**Authors:** Wei-Ming Cheng, Ying-Jay Liou, Yu-Hua Fan

**Affiliations:** ^1^Program in Molecular Medicine, School of Life Sciences, National Yang Ming Chiao Tung University, Taipei, Taiwan; ^2^Institute of Biopharmaceutical Science, School of Life Science, National Yang Ming Chiao Tung University, Taipei, Taiwan; ^3^Department of Urology, College of Medicine, National Yang Ming Chiao Tung University, Taipei, Taiwan; ^4^Division of Urology, Department of Surgery, Taipei City Hospital, Zhongxiao Branch, Taipei, Taiwan; ^5^Department of Psychiatry, Taipei Veterans General Hospital, Taipei, Taiwan; ^6^Department of Psychiatry, College of Medicine, National Yang Ming Chiao Tung University, Taipei, Taiwan; ^7^Department of Urology, Taipei Veterans General Hospital, Taipei, Taiwan; ^8^Shu-Tien Urological Research Center, National Yang Ming Chiao Tung University, Taipei, Taiwan

**Keywords:** depression, depression and somatic symptom scale, lower urinary tract symptoms, somatic symptoms, type D personality

## Abstract

This cross-sectional study, which included men aged 20–40 years, aimed to determine the relationships among type D personality, depressive symptoms and lower urinary tract symptoms in young men. An internet-based questionnaire was administered, and General demographics, International Prostate Symptom Scores, Type D Scale-14 scores, and Depression and Somatic Symptom Scale scores were analyzed. A total of 3,127 men were included; of these, 762 (24.4%) reported moderate/severe lower urinary tract symptoms, and 1,565 (50.05%) met the criteria for type D personality. Men with type D personality had significantly higher body mass index and total and sub-scores for the International Prostate Symptom Score and Depression and Somatic Symptom Scale. Furthermore, the type D personality group had a higher prevalence of lower urinary tract symptoms, particularly voiding symptoms. Univariate analysis revealed that all parameters, except for body mass index, were significant predictors of moderate/severe lower urinary tract symptoms. Multivariate analysis showed that age >30 years, type D personality, and depressive and somatic Depression and Somatic Symptom Scale sub-scores were independent predictors of moderate/severe lower urinary tract symptoms. Regarding Type D Scale-14 subscales, social inhibition, rather than negative affectivity, impacted moderate/severe lower urinary tract symptoms. Mediation analysis revealed that depressive symptoms mediated the relationship between type D personality and lower urinary tract symptoms. This study established correlations between type D personality, depressive symptoms, and lower urinary tract symptoms. As previous studies suggested that patients with type D personality are less likely to consult and adhere to treatment, and are at higher risk for depression, urologists should therefore actively recognize patients with TDP.

## Introduction

Lower urinary tract symptoms (LUTS) in men commonly manifest as storage, voiding, and post-micturition symptoms, mainly affecting the bladder, prostate, and urethra. LUTS have been reported to significantly impact an individual's quality of life (Kim et al., 2015), resulting in distress and reduced confidence, further affecting work-related productivity and social interaction. LUTS mainly occur in the elderly; the prevalence of LUTS has been estimated in several large population-based studies from various regions of the world. In Western Europe and North America, the Epidemiology Urinary Incontinence and Comorbidities (EPIC) study, a telephone survey in Canada, Germany, Italy, Sweden, and the UK, reported that the prevalence of at least one LUTS was 62.5 and 66.6% in men and women aged ≥40 years, respectively (Irwin et al., 2006). In Asia, an internet survey with participants from China, Taiwan, and South Korea reported the prevalence of LUTS of 62.8% in men and 59.6% in women aged ≥40 years (Chapple et al., 2017). In South America, the Brazil LUTS, a telephone interview conducted in five major cities of Brazil, indicated that LUTS affected 69% and 82% of men and women aged ≥40 years, respectively (Soler et al., 2018). However, LUTS also affect young adults; for example, a study involving South Korean men aged 19–39 years reported that 28.7% had mild-to-severe LUTS (Kim et al., 2019).

Systemic diseases, including psychiatric diseases, can also cause LUTS, and the bidirectional association between depression and LUTS has been well-established. A secondary analysis of the case-control data from the EPIC study revealed that participants with overactive bladder (OAB)/storage LUTS reported significantly higher rates of depressive symptoms (Center for Epidemiologic Studies Depression Scale, CES-D, ≥21) compared with demographically matched controls (11.4 vs. 3.6%) (Coyne et al., 2008). A population-based study in South Korea reported that 11.5% of individuals with LUTS had depressive symptoms (CES-D ≥ 21) compared with 2.9% without LUTS (Kim et al., 2015). A Chinese longitudinal study showed that men with moderate/severe LUTS were 2.5 times more likely to have depressive symptoms (Geriatric Depression Scale, GDS, ≥ 8) than men with mild or no LUTS (Chung et al., 2013). The Boston Area Community Health (BACH) survey, a community-based epidemiologic survey, found that LUTS were significantly associated with depression (CES-D ≥ 21), and that depression increased the odds of LUTS (Kupelian et al., 2009). Breyer et al. (2014) examined the association of LUTS with suicidal ideation in 2,890 men who were 40 years old or older and reported that men with greater depression scores (Patient Health Questionnaire-9, PHQ-9) were more likely to suffer from LUTS. Huang et al. (2017) conducted a nationwide population-based cohort study using claims data obtained from the Taiwan National Health Insurance Research Database to examine the association between LUTS and anxiety/depression, and found that patients with depression were 2.37 times more likely to develop LUTS. We believe that the association between depression and LUTS would be stronger among young adults because they have a lower incidence of benign prostate hyperplasia and bladder dysfunction, which are common causes of LUTS.

Type D (distressed) personality (TDP) is a broad personality type characterized by the simultaneous occurrence of negative affectivity (NA) and social inhibition (SI), as described by Denollet (2005). NA refers to the tendency to experience negative emotions, such as anger or anxiety, depending on the situation (Watson and Pennebaker, 1989). SI pertains to the tendency to inhibit expressions of emotions and behaviors during social interactions (Asendorpf, 1993). TDP has been observed to be associated with multiple chronic disorders, particularly cardiovascular diseases (Kupper and Denollet, 2016). Additionally, a relationship between TDP and psychological conditions, such as depression, anxiety, and emotional stress, has been observed (Nefs et al., 2012). Al-Qezweny et al. (2016) reported that percutaneous coronary intervention-treated patients with TDP had a 3.69-fold increased risk for depression (Hospital Anxiety and Depression Scale, HADS, ≥8) at 10 years of follow-up.

Studies have shown a strong bidirectional relationship between LUTS and depression. TDP was reported to be independently associated with depression. However, the relationship between LUTS and TDP is not explored. Furthermore, people with TDP have the tendency to experience negative emotions, which might impact the occurrence of LUTS in daily life. Individuals with TDP also tend to avoid social interaction, leading to bothersome and exacerbated LUTS in public toilets (also called paruresis) (Boschen, 2008). Therefore, this study aimed to investigate the isolation and interactive relationship between TDP, depression and LUTS. We hypothesized that TDP causes LUTS and the relationship between TDP and LUTS is mediated by depression.

## Materials and Methods

### Study Design and Population

This was a cross-sectional, population-representative, internet-based survey conducted between February 1, 2021, and February 28, 2021. This study included adult male participants aged 20–40 years who could read and write in traditional Chinese. A hyperlink to the questionnaire was provided to adult Facebook users who satisfied the criteria. Proprietary algorithms and browser fingerprinting technology involving IP address recognition were applied to prevent multiple responses by the same person using different user credentials. Additionally, resubmission was prevented via the inclusion of a verification question at the beginning of the questionnaire. To protect the identity of responders, data were presented as numerical identification for the analysis. The study was approved by the Ethics Committee of Taipei City Hospital (TCHIRB-10911002-E) who confirmed that no written informed consent from participants included in this study was warranted.

### Questionnaires

The questionnaire comprised three parts. The first part collected data regarding general demographics, including age and body mass index (BMI). Second, LUTS were assessed using the International Prostate Symptom Score (IPSS) questionnaire. Third, TDP and depression were assessed using the Type D Scale-14 (DS14) and Depression and Somatic Symptom Scale (DSSS), respectively.

BMI is calculated as weight in kilograms divided by the square of the height in meters (kg/m^2^) and is categorized into four groups according to the Asian-Pacific cutoff points: underweight (<18.5 kg/m^2^), normal weight (18.5–22.9 kg/m^2^), overweight (23–24.9 kg/m^2^), and obese (≥25 kg/m^2^) (Pan and Yeh, 2008).

The IPSS comprises seven questions relating to symptoms of voiding (incomplete emptying, intermittency, weak stream, and straining to void) or storage (frequency, urgency, and nocturia). Each question is graded on a scale of 0–5 (0: not at all, 5: almost always). A total score of 0–7 indicates mild symptoms; 8–19, moderate symptoms; and 20–35, severe symptoms. The voiding and storage sub-scores were calculated separately; additionally, the voiding-to-storage (V/S) sub-score ratio was calculated. V/S is a simple and useful tool to differentiate failure to void and failure to store lower urinary tract dysfunction in men with LUTS. Liao et al. (2011) reported that failure to void was observed in 81.2% of patients with V/S scores > 1, while failure to store was observed in 75.7% of patients with V/S scores ≤ 1.

TDP was assessed using the revised Taiwanese version of the DS14 (DS14-TR) (Denollet, 2005; Weng et al., 2013), which is a 14-item questionnaire that is equally divided into subscales measuring NA and SI. Items are scored on a 5-point Likert scale ranging from 0 (false) to 4 (true), with each subscale score ranging from 0 to 28. A score > 10 on both subscales is classified as TDP.

The DSSS has been validated for use in patients with major depressive disorder in Taiwan. It comprises a 12-item depression subscale and a 10-item somatic subscale (Hung et al., 2006). Each item is scored from 0 to 3 (absent, mild, moderate, and severe, respectively) according to the severity of symptoms, with the total score ranging from 0 to 36 and 0 to 30 for the depression and somatic subscales, respectively.

### Statistics

Descriptive statistics were performed based on two strata represented by the presence and absence of TDP. The chi-squared test was used to compare the frequency of nominal variables, whereas the independent Student's t-test was used to compare the means of continuous variables. Univariate and multivariate logistic regression analyses were conducted to determine the odds ratios and 95% confidence intervals to identify factors related to moderate/severe LUTS (defined as IPSS ≥8). Variables with a *P* < 0.05 in the univariate analysis were included in the multivariate model.

**Figure 1:**
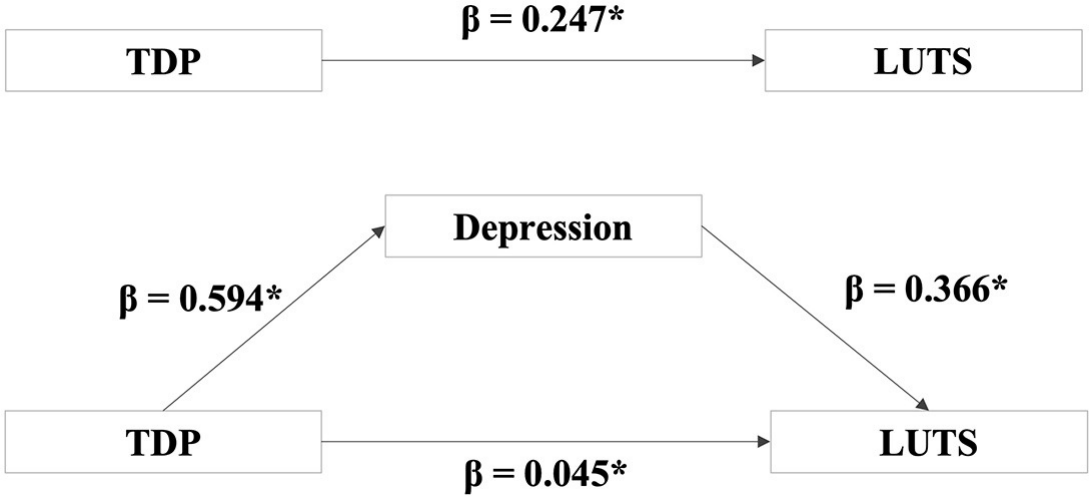
Depression partially mediated the relationship between TDP and LUTS. After controlling for depression, it was observed that the direct effect between TDP on LUTS was still statistically significant (β = 0.045, *P* = 0.01) but less than the direct effect without controlling depression (β = 0.247, *P* < 0.00). β = the regressive parameter estimate. *P < 0.05.

The mediation model was tested using Baron and Kenney's multistage regression approach (Baron and Kenny, 1986). Figure 1 shows the hypothesized mediation model. Four regression equations were generated. In the first equation, a simple regression analysis with TDP as measured by the DS14-TR predicting LUTS as measured by IPSS was conducted. In the second equation, the depression as measured by the summed score of the DSSS was regressed on TDP. In the third equation, LUTS was regressed on depression. Lastly, LUTS was regressed on both, the depression and TDP, accordingly. Full mediation occurs if the effect of the independent variable (TDP) on dependent variable (LUTS) controlling for the mediator (depression) is zero. If the effect of the independent variable on the dependent variable is less but still significant in the last equation, the model can be considered as a partial mediation model (Baron and Kenny, 1986).

All the statistical analyses were performed using the IBM SPSS Statistics software package for Mac version 24 (IBM Corp., Armonk, NY, USA). Statistical significance was set at *P* < 0.05 (two-sided).

## Results

A total of 3,127 men aged 20–40 years responded to the questionnaire; of these, 1,565 (50.05%) were classified as having TDP. The demographic characteristics and questionnaire responses of all participants are summarized in Table 1. The BMI of men with TDP was significantly higher than that of men without TDP (25.1 ± 4.8 vs. 24.8 ± 4.4 kg/m^2^, *P* = 0.049). Nevertheless, the prevalence of underweight, normal weight, overweight and obesity were similar between men with TDP and those without TDP. The IPSS, including the total scores, voiding, and storage sub-scores, was significantly higher in men with TDP (all *P* < 0.001). Men with TDP had a significantly higher prevalence of moderate/severe LUTS than men without TDP (32.1 vs. 16.6%, *P* < 0.001). Moreover, men with TDP exhibited a more prevalent V/S ratio of > 1 (44.0 vs. 39.7%, *P* = 0.017), indicating more profound voiding symptoms. Additionally, men with TDP had a significantly higher DSSS total score and depressive and somatic sub-scores than men without TDP (all *P* < 0.001).

**Table 1:** Demographic description and questionnaire survey of the study participants.

	**Total (*n* = 3,127)**	**Non-type D (*n* = 1,562)**	**Type D (*n* = 1,565)**	***P* value**
Age, years old	30.7 ± 5.5	30.7 ± 5.5	30.6 ± 5.5	0.6
BMI	25.0 ± 4.6	24.8 ± 4.4	25.1 ± 4.8	0.049
BMI categories				0.663
Underweight, *N* (%)	118 (3.8)	61 (3.9)	57 (3.6)	
Normal, *N* (%)	1,033 (33.0)	515 (33.0)	518 (33.1)	
Overweight, *N* (%)	694 (22.2)	359 (23.0)	335 (21.4)	
Obesity, *N* (%)	1282 (41.0)	627 (40.1)	655 (41.9)	
**IPSS**				
Total	5.2 ± 5.2	4.1 ± 4.3	6.3 ± 5.8	<0.001
Voiding	2.6 ± 3.4	1.9 ± 2.7	3.3 ± 3.8	<0.001
Storage	2.7 ± 2.4	2.3 ± 2.2	3.1 ± 2.6	<0.001
**LUTS**				<0.001
Mild, *N* (%)	2,365 (75.6)	1,303 (83.4)	1,062 (67.9)	
Moderate, *N* (%)	687 (22.0)	244 (15.6)	443 (28.3)	
Severe, *N* (%)	75 (2.4)	15 (1.0)	50 (3.8)	
**IPSS-V/S**				0.017
> 1, *N* (%)	1,308 (41.8)	620 (39.7)	688 (44.0)	
≤ 1, *N* (%)	1,819 (58.2)	942 (60.3)	877 (56.0)	
**DS14**				
Total	24.2 ± 11.8	14.8 ± 7.1	33.6 ± 7.1	<0.001
NA	12.7 ± 6.9	7.9 ± 5.3	17.5 ± 4.7	<0.001
SI	11.5 ± 6.2	7.0 ± 4.6	16.1 ± 3.8	<0.001
**DSSS**				
Total	11.7 ± 10.0	7.4 ± 6.7	16.0 ± 10.8	<0.001
Depression	7.1 ± 6.2	4.2 ± 4.1	10.0 ± 6.6	<0.001
Somatic	4.6 ± 4.6	3.2 ± 3.4	6.0 ± 5.2	<0.001

*All values given as mean ± SD and number (%).*

*BMI, body mass index; IPSS, international prostate symptom score; LUTS, lower urinary tract symptoms; V/S, voiding-to-storage subscore ratio; DS14, Type D Scale-14; NA, negative affectivity; SI, social inhibition; DSSS, depression and somatic symptom scale*.

Logistic regression analysis was performed to further clarify the relationships among moderate/severe LUTS, depressive symptoms, and TDP (Table 2). Univariate analysis revealed that all parameters, except for BMI, were statistically significant predictors of moderate/severe LUTS (all *P* < 0.001). The first multivariate model included age, NA and SI sub-scores, and depressive and somatic sub-scores of the DSSS; among them, only the NA sub-score was not a significant independent variable predicting moderate/severe LUTS. The second multivariate model included age, presence of TDP, and depressive and somatic sub-scores of the DSSS; all parameters were significant independent predictors of moderate/severe LUTS.

**Table 2:** Logistic regression analyses of variables associated with moderate/severe LUTS among participants.

**Variable**	**Crude OR (95% CI)**	***P*-value**	**Adjusted OR (95% CI)**	***P*-value**	**Adjusted OR (95% CI)**	***P*-value**
**Age**						
<30	1		1		1	
≥ 30	1.522 (1.290–1.794)	<0.001	1.528 (1.283–1.820)	<0.001	1.514 (1.272–1.802)	<0.001
**BMI**						
<25	1					
≥ 25	0.993 (0.841–1.173)	0.936				
**DS14**						
Total	1.047 (1.039–1.055)	<0.001				
NA	1.079 (1.065–1.092)	<0.001	1.013 (0.994–1.033)	0.181		
SI	1.070 (1.056–1.085)	<0.001	1.018 (1.000–1.036)	0.047		
**TDP**						
No	1				1	
Yes	2.383 (2.009–2.825)	<0.001			1.398 (1.149–1.700)	<0.001
**DSSS**						
Total	1.069 (1.060–1.078)	<0.001				
Depression	1.110 (1.095–1.125)	<0.001	1.065 (1.041–1.090)	<0.001	1.072 (1.052–1.094)	<0.001
Somatic	1.129 (1.110–1.149)	<0.001	1.048 (1.023–1.073)	<0.001	1.046 (1.021–1.072)	<0.001

*LUTS, lower urinary tract symptoms; BMI, body mass index; DS14, type D Scale-14; NA, negative affectivity; SI, social inhibition; TDP, type D personality; DSSS, depression and somatic symptom scale*.

The overall results of the mediation model are shown in Figure 1. After controlling for the depression, it was found that the direct effect of TDP on the LUTS was still statistically significant (β = 0.045, *P* = 0.01) but less than the direct effect without controlling the depression (β = 0.247, *P* < 0.001). This finding supported a partial mediation model, suggesting that TDP affects LUTS, both directly as well as through depression.

## Discussion

This study aimed to evaluate the relationships among TDP, depression, and LUTS in young men. We observed that 50% of men aged 20–40 years had TDP, and that TDP was associated with increased BMI, increased depressive and somatic symptoms, and increased severity of LUTS, particularly voiding symptoms. Age >30 years, higher depressive and somatic sub-scores of the DSSS, and presence of TDP were independent predictors of moderate/severe LUTS. However, among the characteristics of TDP, SI was a better predictor of moderate/severe LUTS than NA. Furthermore, depression partially mediated the association between TDP and LUTS.

The overall prevalence of TDP has been reported to range from 16.6 to 38.5% (Mols and Denollet, 2010). Borkoles et al. (2010) reported that 164 (29%) of 564 British men aged 18–55 years had TDP. Kunst et al. (2009) investigated 154 (111 men and 40 women) aged 22–59 years Dutch prison workers and indicated that 16.6% of the participants had TDP. Polman et al. (2010) conducted a study in 334 (180 men and 154 women) UK university students to explore the relationship between TDP and stress; they reported that 24.9% of participants were classified as TDP. In this study, the prevalence of TDP was relatively higher than that reported by previous studies at approximately 50%. The difference in the prevalence of TDP may be attributable to differences in in the distribution of age, race, or gender ratio. Moreover, general volunteer bias inherent in several survey studies is also a factor to consider, and our sample was obtained from Facebook users who may have different personality traits compared with the general population. Sindermann et al. (2020) indicated that Facebook users reported higher levels of extraversion and lower levels of conscientiousness than non-users. Additionally, a longitudinal study showed a bidirectional relationship between social media use and neuroticism (Andrews et al., 2020).

Few studies have investigated the association between personality traits and LUTS. Klasa et al. (2019) reported the association of neurotic personality traits with the presence of psychogenic LUTS. A study conducted by Koh et al. (2014) on South Korean male patients with LUTS suggestive of prostatic hyperplasia revealed an association between treatment outcomes and personality traits. Neuroticism was associated with a significantly worse treatment response; in contrast, extraversion was associated with a significantly better treatment response, and openness was associated with a high responder rate. Nevertheless, to the best of our knowledge, our study is the first to show the relationship between LUTS and TDP—that is, increased LUTS severity was associated with TDP. TDP was an independent predictor of moderate/severe LUTS. Among the components of TDP, SI alone was a predictor of moderate/severe LUTS. Furthermore, TDP affected LUTS, both directly as well as through depression.

However, no direct evidence regarding the relationship between SI and LUTS was observed. Nevertheless, a few studies have reported that individuals with autism spectrum disorder (ASD), characterized by difficulties in communication and social interaction, tend to experience LUTS. von Gontard et al. (2015) reported a higher prevalence of LUTS, particularly urgency and postponement, in children with ASD. Gubbiotti et al. (2019) revealed that young and adult patients with ASD have a higher prevalence of bladder dysfunction, particularly urinary incontinence. Moreover, an association between SI and heightened sympathetic activation was established (Duijndam et al., 2020). Oh et al. (2013) suggested that an imbalance in autonomic nervous system activity may be a factor that evokes different LUTS in men. Choi et al. (2010) observed increased sympathetic activity in patients with voiding symptom-predominant LUTS compared with storage symptom-predominant LUTS. Additionally, our study revealed that participants with TDP had a higher ratio of voiding symptoms. These data provide evidence on the indirect association between SI and LUTS development.

NA, the other TDP personality characteristic examined, is considered a vulnerability factor for depressive symptoms and has been reported to be a cardinal symptom of major depressive disorder in a previous study (Iqbal and Dar, 2015). Our study showed a strong relationship between the severity of depressive symptoms and moderate/severe LUTS. Theoretically, NA should be a predictor of moderate/severe LUTS; however, we established no relationship between NA and moderate/severe LUTS after adjusting for depressive and somatic symptoms. NA is a broad personality trait that refers to the tendency to experience negative emotions. Individuals with depression and high levels of NA are more likely to experience negative affective mood states across different situations (Watson and Pennebaker, 1989). The lack of correlation between NA and LUTS may be attributable to the less severe NA in patients with TDP alone, compared with that in patients with depression.

Some evidence suggests a correlation between TDP and an unhealthy lifestyle, including less regular exercise and consumption of unhealthy foods. Physical inactivity, decreased vegetable and increased meat consumption, greater total energy intake, and higher sodium intake are risk factors for LUTS development (Maserejian et al., 2009; Park et al., 2018). Furthermore, patients with TDP are less likely to consult medical specialists and are poorly adherent to medications (Nefs et al., 2015; Li et al., 2016). Based on these findings, the participants with TDP and moderate/severe LUTS in our survey might be reluctant to visit urologists for further evaluation and treatment, resulting in persistent and progressive symptoms.

Studies about personality and BMI associations have mainly focused on broad personality traits (Buratta et al., 2020). Few studies have explored the association between TDP and BMI. The present findings suggested that TDP was associated with increased BMI with borderline significance (*P* = 0.049) and the prevalence of each category of BMI was similar between men with TDP and those without TDP. A study conducted by Lin et al. (2020) on Taiwanese patients with type 2 diabetes mellitus reported that the distributions of BMI categories were not different between patients with TDP and those without TDP. Shao et al. (2017) investigated 532 Chinese patients with type 2 diabetes mellitus and revealed that TDP was not related to increasing BMI. In a cross-sectional Dutch community study exploring the relation between TDP and metabolic syndrome, Mommersteeg et al. (2010) found that there was no difference in waist-circumference or obesity between the Type D and the non-Type D group. Currently available data do not support the association between TDP and BMI.

This study has some limitations. First, a cross-sectional design was used to analyze the association between TDP, depressive symptoms, and LUTS, which may limit conclusions regarding causality. Therefore, a longitudinal study is warranted to understand the direction of the relationship. Furthermore, the influence of TDP on LUTS treatment response and the impact of interventions for TDP on LUTS remain unknown. A future prospective study can help clarify these questions and improve the care of patients with LUTS. Second, the study focused on young men aged 20–40 years, which may limit the generalizability of results. Third, we used Facebook to recruit the study participants; therefore, the sample might not be representative of the general population. Fourth, the limited length of the questionnaire required the exclusion of some information, including comorbidities, prostate size, and medication history. Therefore, we targeted young adult men because they had a low prevalence of physical comorbidities and a low possibility of long-term medication use. The impact of psychiatric conditions on LUTS would be more profound than that of physical comorbidities.

To the best of our knowledge, this study is the first to examine and show a significant association between TDP and LUTS in a large population using well-established and validated assessments. As such, our results require replication in other populations and settings.

In conclusion, our study revealed that 24.4% of men aged 20–40 years had moderate/severe LUTS. TDP was an independent predictor of moderate/severe LUTS. Regarding the components of TDP, SI was a more effective predictor of moderate/severe LUTS than NA. Men with TDP experienced significantly higher levels of depressive symptoms. TDP affected LUTS, both directly as well as through depression. Because patients with TDP are less likely to consult medical specialists, are more likely to be poorly adherent to medications, and are at higher risk for depression, urologists should therefore actively recognize patients with TDP.

## Data Availability Statement

The raw data supporting the conclusions of this article will be made available by the authors, without undue reservation.

## Ethics Statement

The studies involving human participants were reviewed and approved by Taipei City Hospital. Written informed consent for participation was not required for this study in accordance with the national legislation and the institutional requirements.

## Author Contributions

W-MC: design of the work, acquisition of data, and analysis and interpretation of data. Y-JL: conception of the work. Y-HF: conception of the work, analysis and interpretation of data, and drafting the work. All authors contributed to the article and approved the submitted version.

## Funding

This work was supported by the Taipei Veterans General Hospital under Grant V110C-209. The funding source had no role in the design, practice or analysis of this study.

## Conflict of Interest

The authors declare that the research was conducted in the absence of any commercial or financial relationships that could be construed as a potential conflict of interest.

## Publisher's Note

All claims expressed in this article are solely those of the authors and do not necessarily represent those of their affiliated organizations, or those of the publisher, the editors and the reviewers. Any product that may be evaluated in this article, or claim that may be made by its manufacturer, is not guaranteed or endorsed by the publisher.
